# Effective dose of propofol combined with a low-dose esketamine for gastroscopy in elderly patients: A dose finding study using dixon’s up-and-down method

**DOI:** 10.3389/fphar.2022.956392

**Published:** 2022-09-20

**Authors:** Yuling Zheng, Yafei Xu, Bixin Huang, Ying Mai, Yiwen Zhang, Zhongqi Zhang

**Affiliations:** ^1^ Department of Anesthesiology, the Affiliated Shunde Hospital of Jinan University, Foshan, China; ^2^ Department of Anesthesiology, Shunde Hospital of Southern Medical University, Foshan, China

**Keywords:** propofol, esketamine, effective dose, gastroscopy, dose-response relationship

## Abstract

**Objective:** This study aimed to determine the optimal dose of propofol combined with esketamine to inhibit the response to gastroscope insertion in elderly patients.

**Methods:** This is a prospective, non-controlled, non-randomized, single-center study. Elderly patients aged 65–80 years were enrolled in the study with the American society of anesthesiologists (ASA) physical status I or II undergoing elective gastroscopy. All patients were administered propofol after an intravenous esketamine at the dosage of 0.3 mg/kg 30 s, the subsequent dose of propofol was determined by the response of the previous patient to gastroscope insertion (choking, body movement, etc.) using Dixon’s up-and-down method. The initial dose of propofol administered to the first elderly patient was 3.0 mg/kg, and the standard ratio of propofol dose in adjacent patients was 0.9. At least six crossover points were obtained before the conclusion of the study. By using Probit analysis the median effective dose (ED_50_), 95% effective dose (ED_95_), and the corresponding 95% confidence interval (CI) for propofol were determined.

**Results:** The study continued until we obtained seven crossover points and 32 elderly patients (17 males and 15 females) were collected. The ED_50_ of propofol combined with esketamine inhibiting response to gastroscope insertion in elderly patients were found to be 1.479 mg/kg (95% CI 1.331–1.592 mg/kg), and ED_95_ was found to be 1.738 mg/kg (95% CI 1.614–2.487 mg/kg).

**Conclusion:** According to the present study, propofol combined with 0.3 mg/kg esketamine is safe and effective for elderly patients undergoing gastroscopy. The ED_50_ and ED_95_ doses of propofol inhibiting response to gastroscope insertion in elderly patients when combined with 0.3 mg/kg esketamine were 1.479 and 1.738 mg/kg, respectively, without apparent adverse effects.

## 1 Introduction

During upper gastrointestinal endoscopy, the endoscope probe stimulates the pharynx when it enters the esophagus. As the pharynx is highly sensitive, patients tend to have nausea, vomiting, choking cough, and even laryngospasm, which can be considerably reduced with an adequate painless procedure. Appropriate sedation enables patients to pass the examination process without difficulty and improves the completion rate and accuracy of endoscopic examination with an enhanced patient and endoscopist satisfaction (Padmanabhan et al., 2017; Nishizawa and Suzuki, 2018). Propofol is a short-acting sedative-hypnotic drug with the characteristics of rapid onset of action, quick recovery, and fewer adverse effects that have been widely used in painless digestive endoscopy (Padmanabhan et al., 2017). Nevertheless, propofol has been reported to possess dose-dependent adverse effects, where overdose increases the risk of respiratory and circulatory depression, while underdose causes airway irritation, pain, limb twitching, and even gastroscopy interruption (Vaessen and Knape, 2016; Delgado et al., 2019), mostly in older patients. In many cases, adjuvants are required because co-administration could not only decrease the required dose of propofol but also reduces the incidence of adverse drug reactions (Nam et al., 2022).

Esketamine is the s-enantiomer of ketamine, and its anesthetic effect is approximately threefold that of R (-) -ketamine (Zanos et al., 2018). Due to the dose-dependent adverse effects of ketamine, low-dose esketamine can decrease the incidence of anesthesia-related adverse events (Bowdle et al., 1998; Zhan et al., 2022). Due to its sympathomimetic properties and less respiratory and circulatory depression, esketamine could better maintain the hemodynamic stability of elderly patients during induction of anesthesia (Yang et al., 2022b; Li et al., 2022). Therefore, it is an ideal candidate to be used in combination with propofol for gastroscopic examination (Wang et al., 2019). However, there is no clear evidence on the effective dose of propofol in combination with esketamine for elderly patients undergoing gastroscopy. Therefore, we conducted this prospective study intending to assess the ED_50_ and ED_95_ of propofol combined with 0.3 mg/kg esketamine for a gastroscopy to inhibit the gastroscope insertion (from the pharynx into the esophagus) reaction in elderly patients, thereby providing clinical advice and medication guidance.

## 2 Materials and methods

### 2.1 Study design and patients

This is a prospective, non-controlled, non-randomized, single-center study. The study was registered at the Chinese Clinical Trial Registry (www.chictr.org.cn; registration number: ChiCTR2000038242) on 15/09/2020. The registration of clinical trial includes different age groups (pediatric group, young-middle-aged group and elderly group) and the present study enrolled only elderly patients (age range from 65 to 80). The study was approved by the Medical Ethics Committee of the Shunde Hospital of Southern Medical University (Number: 20200903). All patients undergoing elective gastroscopy from March to May 2021 were enrolled in the study.

### 2.2 Criteria for inclusion and exclusion

Patients were included if they met the following criteria: age between 65 and 80 years, body mass index (BMI) between 18 and 27 kg/m^2^, perform elective gastroscopy, diagnostic gastroscopy, and ASA physical status I or II.

The following exclusion criteria were implemented in this study: refuse to participate; known allergy to either propofol or esketamine; evident difficult airway; chronic pain; mental-related diseases; symptomatic cardiovascular or pulmonary diseases; severe hepatic and kidney function problems; alcohol abuse; those with increased intracranial pressure or intraocular pressure; history of hyperthyroidism; hemostasis, polypectomy or other require any treatments before/during the examination; chronic use of sedative or analgesic drugs.

### 2.3 Anesthesia protocol and endoscopic procedure

Before the painless gastroscopy, all patients were kept fasted for at least 6 h and were then transferred to the examination room and put on oxygen at the rate of 4 L/min through a nasal straw. The Anesthesiologist also measured the mean arterial pressure (MAP), heart rate (HR), and blood oxygen saturation (SpO_2_) of all patients. After opening the patient’s peripheral venous access, 500 ml of lactated ringer’s solution was infused at a rate of 250 ml/h.

All Anesthesia operations were performed by the same anesthesiologist. Patients were administered intravenous 0.3 mg/kg esketamine (2 ml: 50 mg, Jiangsu Hengrui Medicine China, lot number: 200403BL) and propofol for sedation (20 ml: 0.2 g, Propofol 1% MCT Fresenius Kabi St. Wendel Germany, lot number: 2003085). The dose of propofol administered to elderly patients after 30 s of intravenous esketamine was determined by the response of the previous patient to gastroscope insertion (choking cough, body movement, etc.) using Dixon’s up-and-down method. The dose of propofol was increased in the subsequent patient due to an increase in choking cough, body movement, and effect on operations by endoscopists when the gastroscope inserted into the esophagus was deemed “responsive.” The dose of propofol was decreased in the subsequent patient if there was no choking cough and no body movement. The initial dose of propofol administered to the first elderly patient was 3.0 mg/kg, and the average ratio of propofol dose in adjacent patients was 0.9. At least six crossover points were obtained before the conclusion of the study.

Following the administration of propofol, an endoscope (OLYMPUS Lucera LCV-260SL) was inserted. All gastroscopic examinations were performed by endoscopists with at least five years of experience. If the patient was “responsive”, a single dose of 20–50 mg of propofol was administered intravenously and repeated to complete the gastroscopy.

Following gastroscopy, the patients were transferred to the post-anesthesia care unit (PACU), where the anesthetist awakened the elderly patient. Similarly, the HR, MAP, and SpO_2_ levels were also monitored. The recovery time was recorded. The patient spent at least 30 min in PACU. The criteria for discharge or transfer from PACU to the inpatient unit were typical vital signs, the ability to walk without assistance, and the absence of evident side effects.

The adverse medical events were handled as follows: hypotension (MAP decreased by 30% over the baseline value) was treated with a bolus of 5–10 mg ephedrine; bradycardia (HR < 50 beats/min) was treated with an intravenous injection of 0.25–0.5 mg atropine; respiratory depression (SpO_2_<90%) was treated with a mask pressurized or laryngeal mask to maintain ventilation; and in event of nausea and vomiting, a bolus of 2 mg tropisetron was administered.

### 2.4 Outcome assessments

The primary outcome of the current study was the dose of propofol determined for each elderly patient using Dixon’s up-and-down method.

The secondary outcome: HR, MAP, and SpO_2_ were measured at the following time points: 5 min after entering the gastroscopy room (T1), immediately after intravenous injection of ketamine (T2), immediately after intravenous injection of propofol (T3), and immediately after the Endoscope was passed into the esophagus (T4), and 1 min after the patient’s recovery (T5). Hypotension, bradycardia, injection pain, post-operative nausea and vomiting (PONV), respiratory depression (SpO_2_<90%), emergence agitation, and psychiatric symptoms 24 h following anesthesia were also recorded. Additionally, each dose of propofol used, the duration of the gastroscope insertion and gastroscopy, and the recovery time were recorded. If the patient frowned or complained of arm pain or ipsilateral limb escape response, this was defined as injection pain. Gastroscope insertion time was defined as the duration from the pharynx into the esophagus. Recovery time was defined as the duration between propofol cessation and eye-opening on command.

### 2.5 Statistical analysis

The sample size calculation: The total number of participants depends on Dixon’s up-and-down method (Dixon, 1991). This method requires at least six crossover points (non-responsive to responsive) for statistical analysis.

All the statistical analysis were performed using SPSS (version 17.0, SPSS Inc. Chicago, Illinois, United States). Data were checked for normality using Shapiro-Wilk test, and the appropriate test was next applied as indicated. Data were expressed as mean ± standard deviation (SD), median [range] or *n*. Using repeated measures analysis of variance, the data collected at various time points within the group were analyzed. *p* < 0.05 indicates a statistically significant difference. The ED_50_ and ED_95_ of propofol and their corresponding CI were analyzed using the Probit test. Microsoft excel software was used to draw a sequential graph and dose-response curve.

## 3 Results

### 3.1 Patients information

**FIGURE 1:**
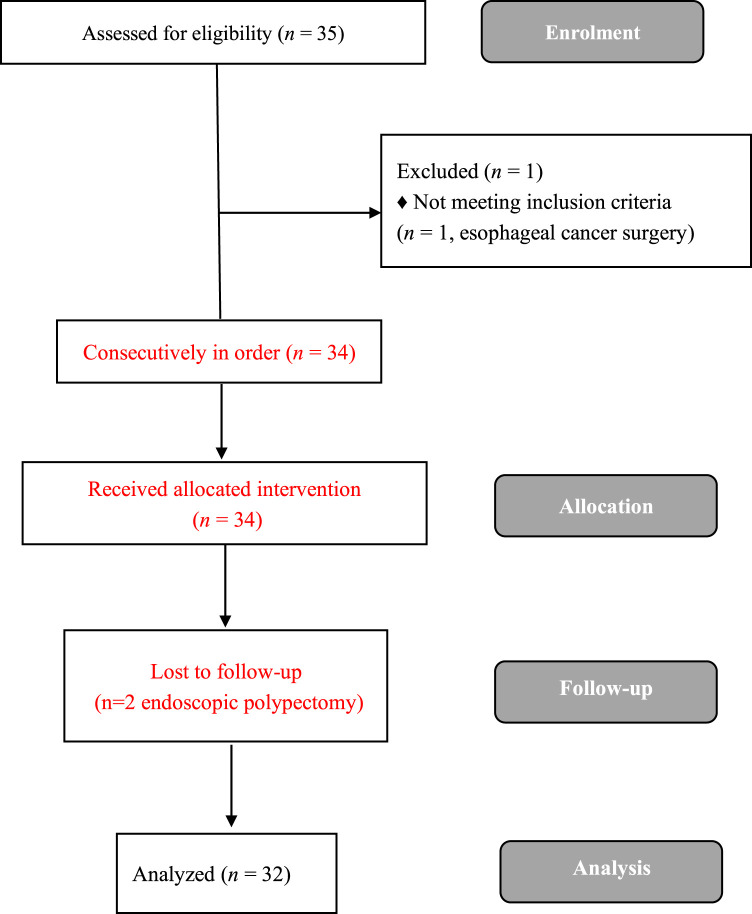
Study flowchart.

A total of 35 elderly patients were enrolled from March to May 2021. Three elderly patients were excluded, and a total of 32 elderly patients successfully completed the study (Figure 1). Table 1 depicts the demographic data of all the elderly enrolled patients. Gastroscopy time was (5.9 ± 1.2) min, recovery time was (11.2 ± 4.3) min, and propofol dose was (119.4 ± 18.3) mg.

**TABLE 1:** Patients’ characteristics.

Index	
Age (years)	66.5 ± 4.0
Gender (F/M)	14/18
BMI (kg/m^2^)	23.9 ± 2.3
ASA status (I/II)	20/12
Smoking (Y/N)	13/19
Drinking (Y/N)	10/22
Hypertension (Y/N)	26/6
Diabetes (Y/N)	14/18
Gastroscope insertion time (s)	6 [5–22]
Gastroscopy time (min)	5.4 ± 1.2
Recovery time **(**min)	11.2 ± 4.3
Dose of Propofol (mg)	119.4 ± 18.3

Values are expressed as mean ± SD, median (range) or number of patients.

### 3.2 Response of gastroscope insertion

**FIGURE 2:**
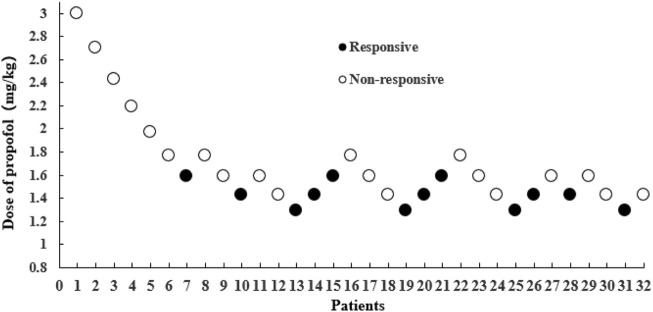
The sequential response of 32 elderly patients to gastroscope insertion with the up-and-down method. The black dot represents “responsive,” and the white dot represents “non-responsive".

Our study was conducted until data of seven crossover points were collected. Figure 2 shows the sequential response of 32 elderly patients to the up-and-down method of gastroscope insertion. There were 12 elderly patients who were responsive and given propofol as a remedy.

### 3.3 ED_50_ and ED_95_ of propofol combined with esketamine

**FIGURE 3:**
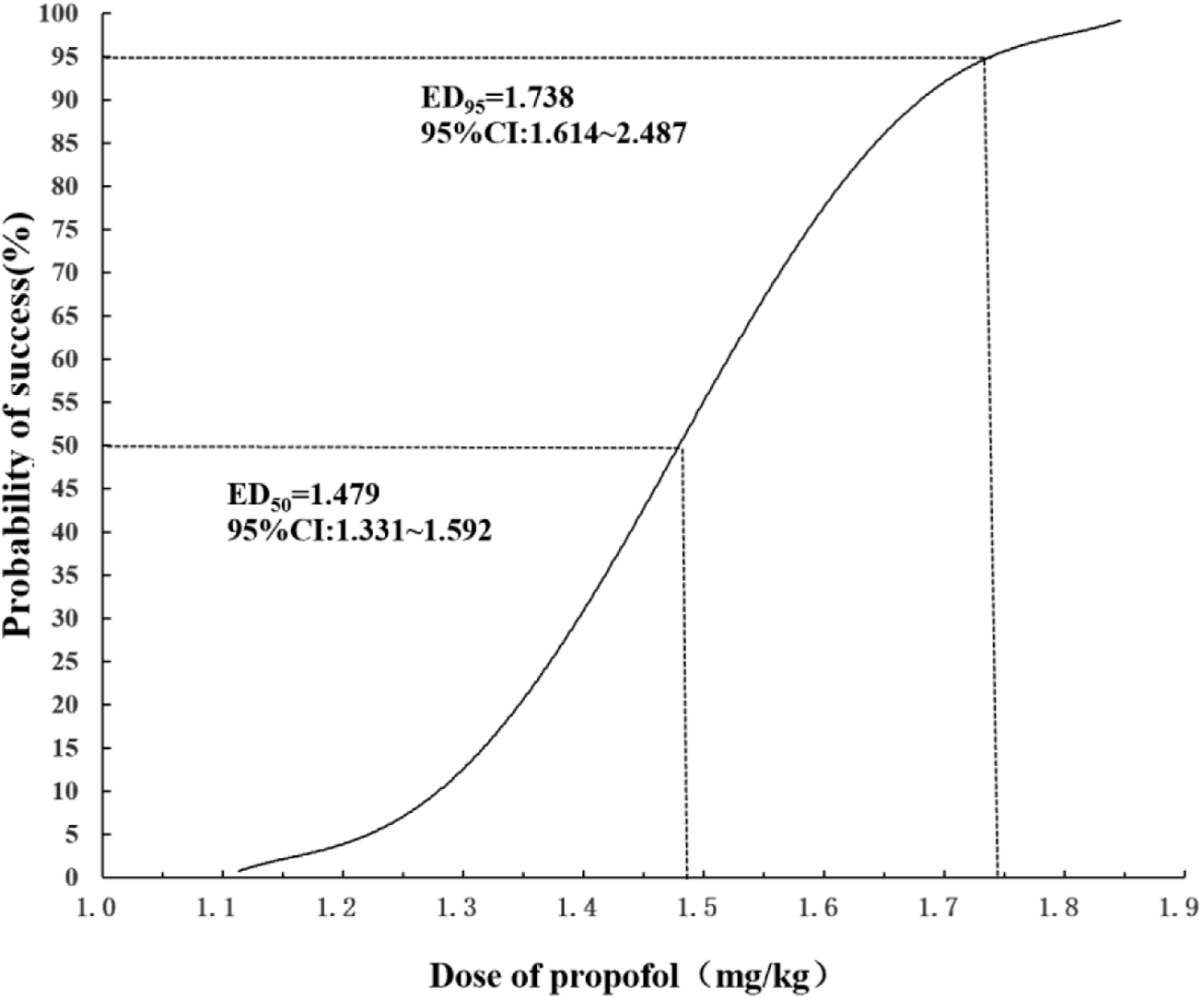
Dose-response curve for propofol plotted using probit analysis.

The ED_50_ of propofol combined with esketamine inhibiting response to gastroscope insertion in elderly patients was found to be 1.479 mg/kg (95% CI: 1.331–1.592 mg/kg), while the ED_95_ was found to be 1.738 mg/kg (95% CI: 1.614–2.487 mg/kg) (Figure 3).

### 3.4 Hemodynamic changes of patients at different time points

According to repeated measures analysis of variance, MAP and HR fluctuated significantly (*p* < 0.05) over time. MAP significantly increased immediately after intravenous injection of ketamine (T2) time point and dropped significantly immediately after intravenous injection of propofol (T3) time point in comparison to 5 min after entering the gastroscopy room (T1) time point without a significant change in HR and SpO_2_ (Table 2).

**TABLE 2:** Hemodynamic changes of patients at different time points.

Items	Timepoint					F	*p*
	T1	T2	T3	T4	T5		
MAP (mmHg)	108.7 ± 14.2	118.3 ± 16.7^*^	90.3 ± 17.9^*^	94.9 ± 14.7	99.1 ± 13.6	36.95	<0.001
HR (beats/min)	77.6 ± 13.5	79.6 ± 12.8	74.6 ± 12.4	75.1 ± 10.9	72.3 ± 11.4	6.46	<0.05
SpO_2_ (%)	99.8 ± 0.4	99.6 ± 0.3	98.1 ± 1.5	99.7 ± 0.5	99.8 ± 0.4	1.59	0.216

Compared with T1, *p < 0.05. Values are expressed as mean ± SD. T1 = 5 min after entering the Gastroscopy room, T2 = immediately after intravenous injection of Ketamine, T3 = immediately after intravenous injection of propofol, T4 = immediately after endoscope passed through the mouth and into the esophagus, and T5 = 1 min after the patient’s recovery (T5).

### 3.5 Anesthesia-related adverse events

The overall incidence of anesthesia-related adverse events was recorded to be 23.3% (Table 3).

**TABLE 3:** Anesthesia-related adverse events.

adverse events	
Hypotension	2
Bradycardia	1
PONV	1
SpO_2_ < 90 %	2
Injection pain	1
Emergence agitation	0
Psychiatric symptoms after 24 h	0
Total	7 (23.3%)

Values are expressed as the number of patients.

## 4 Discussion

The primary objective of this study was to evaluate the effect of low-dose esketamine on the dose of propofol required to achieve the desired degree of sedation without body movement for gastroscopy in elderly patients and its safety and efficacy. Our research revealed that the ED_50_ and ED_95_ values for propofol inhibiting the response to gastroscope insertion in patients when combined with 0.3 mg/kg esketamine were found to be 1.479 and 1.738 mg/kg, respectively. The incidence of anesthesia-related complications was recorded to be 23.3% among all patients. All adverse reactions returned rapidly to normal following treatment, and there was no severe cardiovascular incident during gastroscopy. No significant psychiatric symptoms occurred during the telephone follow-up 24 h after the gastroscopy. Thus, the combinational use of propofol and 0.3 mg/kg esketamine is regarded as safe and effective for elderly patients undergoing gastroscopy.

Similar research evaluated the median effective concentration (EC_50_) of propofol and ketamine in elderly gastrointestinal endoscopy patients (Yang et al., 2022b). To determine the EC_50_ in their study, propofol was administered using a computer-controlled target-controlled infusion (TCI) pump. We believe that the use of TCI has certain advantages for painless gastrointestinal endoscopy. Still, because gastroscopy requires less time than gastrointestinal endoscopy in the present study, direct injection is simple, convenient, and quick, saving anesthesia time and equipment requirements compared to TCI. Thus, direct injection of propofol is appropriate for gastroscopy. The effective dose of propofol obtained using Dixon’s up-and-down method can be utilized to be a better immediate clinically applied medication for gastroscopy.

Like Ketamine, esketamine is an N-methyl-D-aspartate receptor antagonist with analgesic, anesthetic, and sympathomimetic properties and can reduce the incidence of cardiopulmonary depression; however, there is a correlation between effect and dosage, and a small dose of esketamine has greater sedative and analgesic effects than a larger dose (Perez-Ruixo et al., 2021; Yang et al., 2022b; Zheng et al., 2022). Esketamine can be used safely and effectively in elderly patients due to its stable hemodynamics and low incidence of adverse events properties (Yang et al., 2022b). Impaired physical function, the type of surgical procedure, and old age are risk factors for propofol-induced circulatory and respiratory depression (Quine et al., 1995; Eckardt et al., 1999; Bhananker et al., 2006). Therefore, advanced age is also a significant risk factor for adverse events associated with propofol sedation during gastroscopy. Because propofol reduces cardiac output (CO) and systemic vascular resistance (SVR) and causes respiratory depression, it should be administered with caution to elderly patients (Han et al., 2017). After injection of low-dose esketamine, our study revealed that MAP was significantly higher than the baseline blood pressure but had almost no effect on HR and SpO_2_. This transient blood pressure increase could be eliminated by the injection of propofol. Our results were consistent with results reported by Eberl et al, (2020). We believed that it might be due to the sympathomimetic effect of esketamine, which makes it an optimal analgesic and sedative drug for anesthesia in hemodynamically compromised patients. Furthermore, it has been regarded as the drug of choice for elderly patients, in the event of a pre-hospital emergency, burn, and cardiogenic shock patients, as an adjunct to propofol use for sedation (Trimmel et al., 2018; Eberl et al., 2020).

Due to the potential for apnea with propofol, propofol-related respiratory depression occurs frequently reported adverse effects. Ketamine has the unique characteristics of maintaining stable oxygen saturation owing to its ability to exert direct smooth muscle relaxation, bronchodilation effect, and preserve the laryngeal reflex (Cortinez and Anderson, 2018; Iqbal et al., 2022). However, it should be noted that ketamine-induced sedation can also result in the adverse airway and respiratory events, with an incidence of 1.4%–6.6% (Green et al., 2009). In our study, the incidence and severity of hemodynamic and respiratory adverse events were lower than in other reported studies (Akhondzadeh et al., 2016; Bahrami Gorji et al., 2016). Ketamine is a psychoactive drug that can cause neurological and psychiatric complications, including delirium, hallucinations, and dissociative symptoms. None of patients in our study developed psychiatric symptoms during the follow-up by telephone after 24 h. Propofol may inhibit ketamine-induced expression of c-fos in the posterior cingulate cortex, and that overexpression of c-fos leads to psychotomimetic side effects of ketamine (Nagata et al., 1998).

With increasing age, the effective dose range of propofol gradually decreases (Yang et al., 2022a). Researchers estimated that the mean induction dose of propofol for elderly patients was 1.7 mg/kg (Schonberger et al., 2021), which was comparable to the outcomes of our study. Opioids such as remifentanil, sufentanil, or fentanyl combined with propofol were the most frequently used agents in painless gastroenteroscopy, which can reduce the administered dose of propofol (Zhao et al., 2015; Doganay et al., 2017). Compared to propofol alone or in combination with opioids, propofol combined with ketamine provides sedation and analgesia in addition to the reduction in the risk of cardiovascular and respiratory adverse events (Shah et al., 2011; Nazemroaya et al., 2018). It was observed that propofol used in combination with esketamine reduced the therapeutic dose of propofol in comparison to its lone use for producing similar results during gastrointestinal endoscopy in elderly patients. With the increase in esketamine dose (0–0.5 mg/kg), there was no obvious hypotension, and the recovery time was shortened (Yang et al., 2022b). We, therefore, believed that the low incidence of adverse events in this study was associated with a lower propofol induction dose.

The limitations encountered during the study were: First, considering the safety of the drugs in exploratory trials in elderly patients, we only included patients with ASA I or II and excluded high-risk patients with ASA III and IV. Therefore, the results and conclusions of this study may not apply to other high-risk patients. Second, gastroscopies were performed by 3 different operators (with>5 years of experience), and skills and manipulations of gastroscope insertion may affect the results; therefore, these results may have limited applicability. Thirdly, considering that esketamine may have a greater impact on the effect of general anesthesia and hemodynamics in elderly patients, the anesthesiologists were not blinded to the dose of esketamine used, which may have impacted our findings. Consequently, the current findings require further investigation.

## 5 Conclusion

In conclusion, it was discovered that combining 1.738 mg/kg of propofol with 0.3 mg/kg of esketamine inhibits the response to gastroscope insertion in 95% of elderly patients without noticeable adverse reactions. In the future, we aim to further investigate the optimal dose of propofol for elderly patients in combination with other doses of esketamine for gastroscopy.

## Data Availability

The original contributions presented in the study are included in the article/supplementary materials, further inquiries can be directed to the corresponding author.
